# Extracorporeal shock wave therapy does not improve hypertensive nephropathy

**DOI:** 10.14814/phy2.12699

**Published:** 2016-06-02

**Authors:** Jonathan Caron, Pierre‐Antoine Michel, Jean‐Claude Dussaule, Christos Chatziantoniou, Pierre Ronco, Jean‐Jacques Boffa

**Affiliations:** ^1^ INSERM UNIT 1155ParisF‐75020France; ^2^ Université Pierre et Marie Curie‐Paris 6UMR S 1155ParisFrance; ^3^ Department of NephrologyAP‐HPHôpital TenonParisF‐75020France; ^4^ Department of physiologyAP‐HPHôpital Saint‐AntoineParisF‐75012France

**Keywords:** Renal fibrosis, renal repair, angiogenesis, chronic kidney disease

## Abstract

Low‐energy extracorporeal shock wave therapy (SWT) has been shown to improve myocardial dysfunction, hind limb ischemia, erectile function, and to facilitate cell therapy and healing process. These therapeutic effects were mainly due to promoting angiogenesis. Since chronic kidney diseases are characterized by renal fibrosis and capillaries rarefaction, they may benefit from a proangiogenic treatment. The objective of our study was to determine whether SWT could ameliorate renal repair and favor angiogenesis in L‐NAME‐induced hypertensive nephropathy in rats. SWT was started when proteinuria exceeded 1 g/mmol of creatinine and 1 week after L‐NAME removal. SWT consisted of implying 0.09 mJ/mm^2^ (400 shots), 3 times per week. After 4 weeks of SWT, blood pressure, renal function and urinary protein excretion did not differ between treated (LN + SWT) and untreated rats (LN). Histological lesions including glomerulosclerosis and arteriolosclerosis scores, tubular dilatation and interstitial fibrosis were similar in both groups. In addition, peritubular capillaries and eNOS, VEGF, VEGF‐R, SDF‐1 gene expressions did not increase in SWT‐treated compared to untreated animals. No procedural complications or adverse effects were observed in control (C + SWT) and hypertensive rats (LN + SWT). These results suggest that extracorporeal kidney shock wave therapy does not induce angiogenesis and does not improve renal function and structure, at least in the model of hypertensive nephropathy although the treatment is well tolerated.

## Introduction

Ultrasounds are commonly used for medical imaging, but ultrasounds have various therapeutic applications too. Extracorporeal‐generated shock waves were introduced approximately 20 years ago to disintegrate kidney stones. Subsequently, lower energy shock waves have been used in orthopedics and traumatology to treat insertion tendinitis, avascular necrosis of bone. More recently low‐energy extracorporeal shock wave therapy (SWT) has been develop as an angiogenic therapy in chronic diseases (Assmus et al. 2013; Omar et al. 2014). Shock wave is a longitudinal acoustic wave, travelling through body tissue with the speed of ultrasound in water. It is a single pressure pulse with a short needle‐like positive spike <1 *μ*sec in duration and up to 100 MPa in amplitude, followed by a tensile part of several microseconds with lower amplitude (Mittermayr et al. 2012). SW is known to exert the “cavitation effect” and localized stress closed to shear stress. In vitro studies have demonstrated that extracorporeal SWT can enhance vascular endothelial growth factor (VEGF) mRNA expression in cultured human umbilical vein endothelial cells and in bone marrow cells (Aicher et al. 2006; Yip et al. 2008). By applying appropriate energy to organs, SWT can attenuate inflammatory response and induce angiogenesis/vasculogenesis. In humans, SWT is being used in coronary artery diseases (Kikuchi et al. 2010), erectile dysfunction (Vardi et al. 2012), bone fractures (Haupt et al. 1992), calcifying tendinitis (Vulpiani et al. 2009), and diabetic foot ulcers (Assmus et al. 2013).

Chronic kidney disease (CKD) is becoming increasingly common and represents over 13% of general adult population in the US (Coresh et al. 2007). In three large cities in France, two out of seven individuals over 65 years old have an estimated glomerular filtration rate (eGFR) by MDRD less than 60 mL/min/1.73 m^2^ (Stengel et al. 2011). High blood pressure and diabetes represent the two major causes of end‐stage renal disease (ESRD). Moreover, hypertension is a prognosis factor of CKD progression. Interventional studies have emphasized the beneficial effects of ACE inhibitors and angiotensin receptor blockers in reducing systemic and glomerular pressure and urinary albumin excretion, and demonstrated their ability to delay ESRD (The GISEN Group (Gruppo Italiano di Studi Epidemiologici in Nefrologia), 1997; Brenner et al. 2001; Wright et al. 2002). Nonetheless, renal function still declines in most CKD patients (Lewis et al. 2001; Ruggenenti et al. 2008). Renal function decline is associated with development of renal fibrosis and peritubular capillaries rarefaction (Kang et al. 2002). CKD in human is largely a nonreversible process but we were able to demonstrate that hypertensive nephropathy after chronic administration of N^G^‐nitro‐L‐arginine methyl ester (L‐NAME) in rodents could be partly improved at least in two situations. We showed that renal fibrosis could partially regress after L‐NAME removal and reappraisal of nitric oxide in mice (Placier et al. 2006). In a second study, we showed that high dose of angiotensin 2 blocker could improve renal fibrosis induced by L‐NAME‐treated rats (Boffa et al. 2003). Although L‐NAME‐induced hypertension does not mimic human disease, nitric oxide deficiency occurs by multiple mechanisms and contributes to the pathogenesis of hypertension and progression of CKD (Baylis 2012). To improve CKD outcome, additional therapies are needed. After renal injury, several processes are activated to insure healing, restore the initial structure and function. For example, ureteral obstruction induces inflammation, tubular cell atrophy, dilatation, apoptosis, and proliferation, leading to interstitial fibrosis. Relief of the obstruction produces a gradual improvement in renal structure and function, the reappearance of peritubular capillaries, and restoration of renal VEGF content (Bige et al. 2012). Among promising treatments, those favoring angiogenesis might improve renal repair and function. SWT could provide a unique opportunity to develop a new angiogenic therapy for CKD. This study was designed to examine whether SWT could ameliorate renal repair in L‐NAME‐induced hypertensive nephropathy in rats.

## Materials and Methods

### Animal model

NO synthesis was inhibited by administrating N^G^‐nitro‐L‐arginine methyl ester (L‐NAME, 15 mg/kg per day) to male Sprague–Dawley rats (250 g) from Harlan. L‐NAME is a competitive inhibitor of NO synthases (NOS), and therefore its use results in high blood pressure, loss of peritubular microvessels, hypoxia, and renal fibrosis (Zatz and Baylis 1998; Boffa et al. 2003). We added NaCl (6 g/L) in the drinking water to accelerate the model and worsen the severity of renal injury as previously detailed (Fujihara et al. 1994; Ying et al. 2003). All procedures were in accordance with European Union Guidelines for the Care and the use of laboratory animals and were approved the local ethic committee (Comité National de Réflexion Ethique sur l'Expérimentation Animale #05).

**Figure 1:**
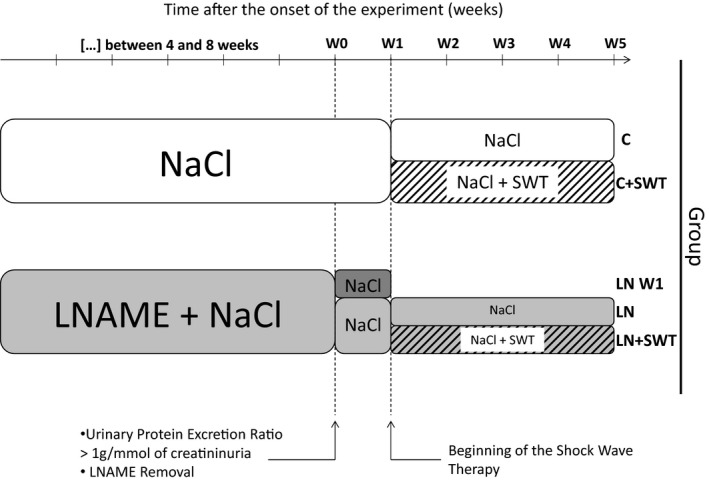
Experimental protocol. When urinary protein excretion ratio was over 1 g/mmol of creatinine, after 6 ± 2 weeks, N^G^‐nitro‐L‐arginine methyl ester (L‐NAME) was removed. Shock wave therapy (SWT) started at W1 for 4 weeks.

Weekly measurement of proteinuria allowed us to set a threshold of 1000 mg/mmol of urinary creatinine beyond which severe nephroangiosclerosis lesions were present and mortality rate was around 20% (Guerrot et al. 2012). At this level of urinary protein excretion, we stopped L‐NAME administration, and monitored the renal repair as previously reported (Placier et al. 2006). The treatment began 1 week later, once the mortality rate was stabilized and renal repair phase initiated. One week after L‐NAME removal, a group of rats was euthanized and studied (LN‐W1) (*n* = 4), other rats previously treated with L‐NAME were divided into two groups: (1) in the LN group (*n* = 14), rats were previously treated by L‐NAME and then allowed to recover for 4 weeks; (2) in the LN + SWT group (*n* = 16), rats were previously treated by L‐NAME and then treated by SWT for 4 weeks. Control rats did not receive L‐NAME. They were randomly divided in two groups: a control group (C) (*n* = 4) and a group treated by SWT (C + SWT) (*n* = 6). All four groups were followed for four additional weeks and euthanized. All of them received NaCl in their drinking water (Fig. 1).

### Extracorporeal shock wave therapy

One week after L‐NAME withdrawal (LN‐W1), we initiated the extracorporeal shock wave therapy (SWT) in the LN + SWT and C + SWT groups. SWT was applied on shortly anesthetized rats with isoflurane, using a specialized focused shock wave probe (Medispec Ltd, Yehud, Israel) at a low‐energy density of 0.09 mJ/mm^2^, onto the left kidney (400 shots), three times a week, for 4 weeks as previously reported (Fujihara et al. 1994). LN + SWT and C + SWT groups were compared with rats anesthetized with the same protocol but which were not treated by SWT. To check the correct delivery of SWT, we were able to visualize the shock wave across the renal tissue by echography performed simultaneously to SWT.

After L‐NAME removal, urinary protein excretion ratio and blood pressure were measured weekly from week 1 (W1), just before the beginning of SWT, to the end of the protocol at week 5 (W5) in all four groups. Renal lesions, plasma urea, and creatinine were assessed in the five groups. At sacrifice, blood sample was collected in an EDTA‐coated tube. Then, heart and kidneys were collected, weighed and split in two halves. One part was snapped‐frozen in liquid nitrogen while the other was fixed in acetic formol alcohol (AFA) solution to be included in paraffin.

### Urinary and blood assay

Urine was collected once per week using metabolic cages and urinary protein excretion ratio (mg protein/mmol creatininuria) was assessed. Plasma urea (mmol/L) and creatinine (μmol/L) were measured using enzymatic and spectrophotometric methods with an automat (Konelab, Thermo Fisher Scientific, Cergy‐Pontoise, France).

### Measurement of systolic arterial pressure

The systolic blood pressure (mmHg) was measured with a tail‐cuff sphygmomanometer (CODA 6+, Kent Scientific Corporation, Torrington, Connecticut). To avoid variations in blood pressure due to day cycle, all measurements were carried out between 9 and 11 am.

### Renal histology and scoring

Fixed samples were paraffin‐embedded using standard procedures, and 3 *μ*m sections were stained with Masson's trichrome. These sections were examined by light microscopy at 200× magnification, and histological lesions including glomerulosclerosis, arteriosclerosis, tubular dilatation, and interstitial fibrosis were evaluated. Glomerulosclerosis was assessed by the percentage of sclerotic glomeruli (ratio of sclerotic glomeruli over normal glomeruli). To quantify arteriosclerosis, a semiquantitative score was used: 1 = normal vessels; 2 = thickened vessels, with a ratio lumen/wall >1; 3 = ratio lumen/wall <1; 4 = artery with obstructed lumen and «onion skin» shape. The percentage of tubular dilatation was also calculated. Finally, an interstitial fibrosis score was used as follow: 1 = no fibrosis; 2 = apparent fibrosis corresponding to less than 10% of the observed field; 3 = extended fibrosis>10% of the observed field. Peritubular capillary density was measured by immunohistochemistry with an antiendothelial antibody (RECA) (ABD Serotec, Oxford, England). The percentage of the field occupied by microvessels positively stained for RECA was calculated with the software analySIS (Olympus, Tokyo, Japan) in 10 representative microscopic fields on each slide.

### Quantitative real‐time PCR

More precise measures of angiogenesis and inflammation were performed with quantitative RT‐PCR. RNA was isolated from kidneys using Trizol (Invitrogen, Camarillo), and was reverse transcribed using reverse transcriptase (Superscript II, Invitrogen, Camarillo). To study angiogenesis, we measured the mRNA expression of VEGF (Vascular Endothelial Growth Factor, major proangiogenic molecule) and its receptor VEGF‐R2 (or KDR, Kinase insert Domain Receptor, principal mediator of VEGF effects), of eNOS (endothelial Nitric Oxide Synthase, producing nitric oxide (NO), thus participating to the angiogenesis process upstream of VEGF pathway) and of HIF‐1*α* (Hypoxia Inducible Factor‐1, central transcription factor of hypoxia response). For the study of inflammation, we measured mRNA expression of SDF‐1 (or CXCL12, Stromal cell‐Derived Factor‐1, chemoattractant of T lymphocytes and monocytes), MCP‐1 (or CCL2, Monocyte Chemoattractant Protein 1, chemoattractant of monocytes and basophiles) and CD‐3 (Cluster of Differentiation 3, specifically expressed by T lymphocytes). To evaluate fibrosis, we assessed mRNA expression of COL3A1 (alpha1 chain of type III collagen, principal component of fibrosis).

### Statistical analysis

Values are expressed as mean ± SEM. Data were analyzed using a student test or ANOVA followed by protected least significant difference Fisher's test of the Graphpad Prism software. Results with *P* < 0.05 were considered statistically significant.

## Results

### Development of L‐NAME‐induced hypertensive nephropathy

**Figure 2:**
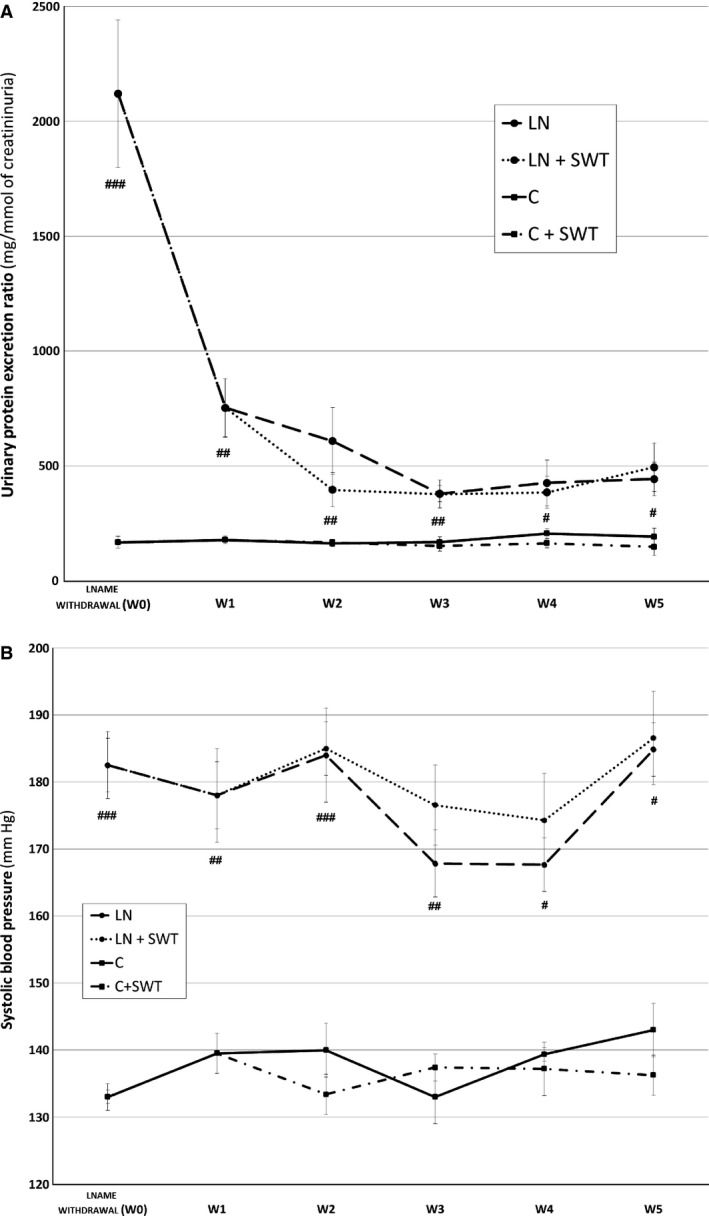
(A) Urinay protein excretion ratio, (B) systolic blood pressure after L‐NAME removal, in controls (C, line with square), controls treated by SWT (C + SWT, broken line with square), L‐NAME‐treated (LN, long broken line with dot) and L‐NAME + SWT‐treated rats (LN + SWT, short broken line with dot). Values are mean ± SEM. ^#^ *P* < 0.05; ^##^ *P* < 0.01, ^###^ *P* < 0.001 versus control; ^§^No significant differences were observed when rats were treated by SWT.

**Figure 3:**
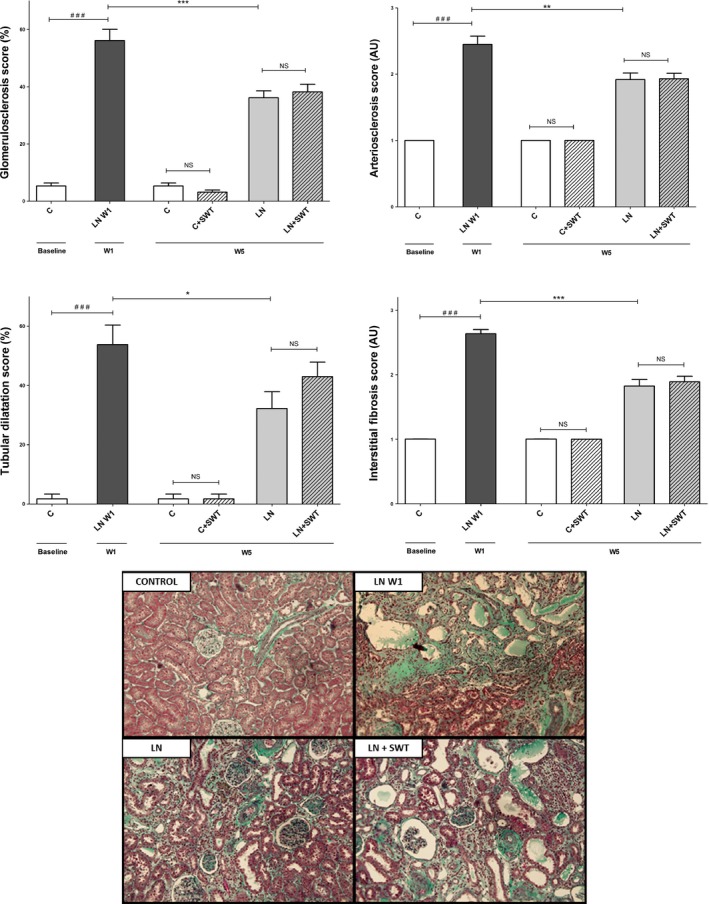
Representative examples of renal tissue (Masson's trichrome) in controls, L‐NAME‐treated rats 1 week after L‐NAME removal (LN‐W1), L‐NAME‐treated + removal rats (LN) and L‐NAME + SWT‐treated rats (LN + SWT) at W5 showing nephroangiosclerosis lesions after NOS inhibition and partial improvement after L‐NAME removal. Quantification of glomerulosclerosis, arteriosclerosis, tubular dilatation, and interstitial fibrosis. All four scores improved after L‐NAME removal but SWT had no effect. ^###^ *P* < 0.001 versus control baseline; *P < 0.05; **P < 0.01, ***P < 0.001 versus LN‐W1; ^§^No significant differences were observed when rats were treated by SWT.

Administration of L‐NAME in the drinking water induced hypertension, high level of urinary protein excretion and typical renal lesions of nephroangiosclerosis (Figs. 2A, B and 3). At L‐NAME removal, urinary protein excretion was 2120 ± 322 mg/mmol, 13‐fold the control baseline group value (157 ± 20 mg/mmol, *P* < 0.001). Systolic blood pressure were 182 ± 5 mm Hg, and 136 ± 6 mmHg, for L‐NAME‐treated rats and control baseline rats, *P* < 0.001. Renal lesions included: (1) glomerulosclerosis characterized by accumulation of hyaline material in the glomerular tuft, adhesion to Bowman's capsule. Other glomeruli were collapsed with enlarged urinary chamber; (2) microvascular lesions ranging from arteriolar wall thickening to onion skin proliferation with complete obliteration and to fibrinoid necrosis of the vascular wall; (3) tubular atrophy and dilatation; and (4) a mild interstitial fibrosis (Fig. 3). Renal function was not altered by L‐NAME administration (not shown).

### Early renal repair after L‐NAME removal

At the early phase of renal repair (W1), urinary protein excretion ratio decreased sharply after L‐NAME removal, from 2120 ± 322 to 753 ± 127 mg/mmol, a higher value than control group, 174 ± 14, *P* < 0.01 (Fig. 2A). In contrast to UPER, hypertension remained stable after L‐NAME removal along the treatment period. At W1, SBP was 178 ± 6 mm Hg for LN‐treated rats, higher than control groups, 141 ± 8 mmHg, p < 0.01 (Fig. 2B).

### Effect of SWT in control rats

At W5, the end of the treatment period, SWT did not change urinary protein excretion, systolic blood pressure and renal function in control rats (Fig. 2A, B; Table 1). All values were similar between C and C + SWT groups. Moreover, renal tissue was not altered by SWT. Glomerulosclerosis and arteriosclerosis index, interstitial fibrosis and tubular dilatation score were similar between C and C + SWT groups suggesting that SWT was safe and well tolerated in control conditions (Table 2).

**Table 1:** Systolic blood pressure and renal function parameters in controls, L‐NAME‐treated rats 1 week after L‐NAME removal (LN‐W1), controls treated by SWT (C + SWT), L‐NAME‐treated + removal rats (LN) and L‐NAME + SWT‐treated rats (LN + SWT) at W5

Function	Control baseline	LN W1	Control	Control + SWT	LN	LN + SWT
	Baseline	W1	W5			
Urinary protein excretion (mg/mmol)	157 ± 20	753 ± 127*	167 ± 21	156 ± 26	443 ± 73	494 ± 105
Systolic blood pressure (mmHg)	136 ± 6	178 ± 6†	134 ± 9	144 ± 10	185 ± 7	187 ± 4
Blood urea nitrogen (mmol/L)	7.19 ± 0.7	7.39 ± 0.7	7.17 ± 0.3	7.18 ± 0.3	7.45 ± 0.5	7.7 ± 0.6
Plasma creatinine (μmol/L)	29 ± 2	30 ± 1	27 ± 1	25 ± 1	47 ± 5***	39 ± 3**

Values are mean ± SEM.

^†^ *P* < 0.05 versus control baseline.

*P < 0.05; **P < 0.01; ***P < 0.001 versus LN‐W1.

**Table 2:** Quantification of renal lesions in controls, L‐NAME‐treated rats 1 week after L‐NAME removal (LN‐W1), controls treated by SWT (C + SWT), L‐NAME‐treated + removal rats (LN) and L‐NAME + SWT‐treated rats (LN + SWT) at W5

Renal lesions	Control baseline	LN W1	Control	Control + SWT	LN	LN + SWT
	Baseline	W1	W5			
% Glomerulosclerosis	5.3 ± 1.1	56 ± 3.9†	5.3 ± 1.1	3.2 ± 0.7	36 ± 2.4***	38 ± 2.6***
Arteriosclerosis score	1	2.5 ± 0.1†	1	1	1.9 ± 0.1**	1.9 ± 0.1***
Interstitial fibrosis score	1	2.6 ± 0.1†	1	1	1.8 ± 0.1***	1.9 ± 0.1***
% tubular dilatation	1.6 ± 1.6	54 ± 7†	1.6 ± 1.6	1.6 ± 1.6	32 ± 6*	43 ± 5

Values are mean ± SEM.

^†^ *P* < 0.001 versus control baseline.

*P < 0.05; **P < 0.01, ***P < 0.001 versus LN‐W1.

### Effects of SWT in previously L‐NAME‐treated rats

Through the treatment period, blood pressure remained high and stable until the sacrifice (Fig. 2B). No difference was observed between LN and LN + SWT groups at W5 (Table 1). At the end of the treatment period, UPER was not different between LN and LN + SWT groups. UPER in these groups tended to be lower than in the LN‐W1 group, but without a significant difference (*P* = 0.14 for LN, *P* = 0.2 for LN + SWT compared with LN‐W1. There is no beneficial effect of SWT on UPER. Plasma urea was similar between LN and LN + SWT at W5, but plasma creatinine rose significantly in both groups during the treatment period compared with LN‐W1, *P* < 0.001. Nevertheless, plasma creatinine was not different between LN and LN + SWT groups at W5 (Table 1). Five weeks after LN removal, although hypertension persisted, renal lesions improved in both LN and LN + SWT groups compared with LN‐W1 group (Table 2; Fig. 3). Repair concerned all compartments of the kidney as glomerulosclerosis and arteriolar scores, interstitial fibrosis and tubular dilatation all decreased significantly. No difference was observed between LN and LN + SWT groups suggesting no effect of SWT on renal repair in L‐NAME‐induced hypertensive rats.

### Peritubular capillaries after L‐NAME administration and withdrawal

**Figure 4:**
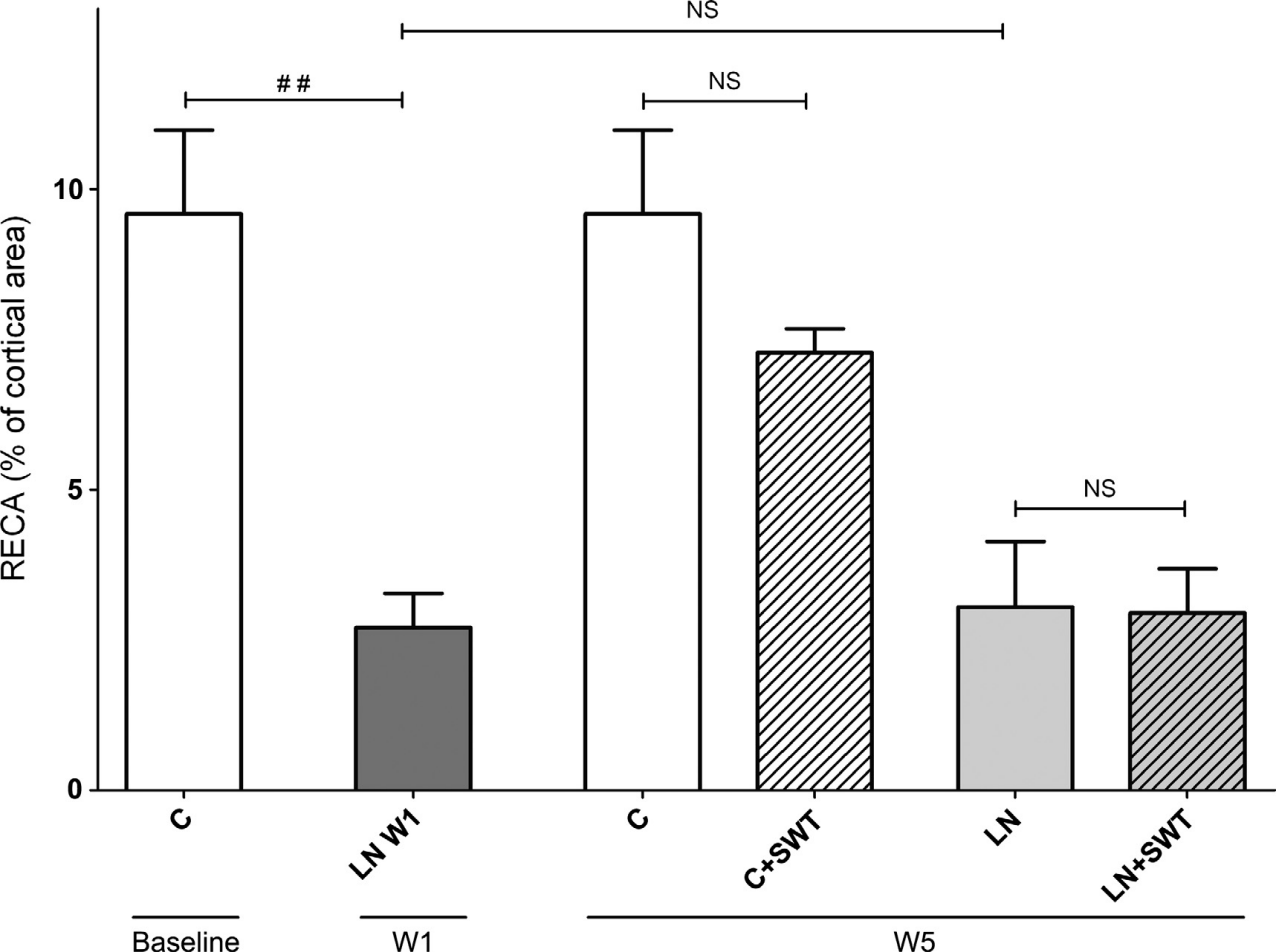
Quantification of RECA‐positive cells by morphometric analysis in controls, L‐NAME‐treated rats 1 week after L‐NAME removal (LN‐W1), L‐NAME‐treated + removal rats (LN) and L‐NAME + SWT‐treated rats (LN + SWT) at W5. The surface area of RECA‐positive cell significantly decreased in L‐NAME‐treated rats. Five weeks after L‐NAME removal, the rarefaction of PTC was sustained and SWT had no effect on peritubular capillaries. ^##^ *P* < 0.01 versus control baseline; ^§^No significant differences were observed when rats were treated by SWT.

The number of peritubular capillaries decreased with the development of nephroangiosclerosis, 2.7 ± 0.6% compared with 9.6 ± 1.4%, *P* < 0.01, respectively, in LN‐W1 and control baseline groups (Fig. 4). L‐NAME removal for 5 weeks did not significantly increase the number of peritubular capillaries (3.1 ± 1.1% in LN group). At W5, SWT did not affect peritubular capillaries in control rats nor in hypertensive rats (Fig. 4).

### Effects of SWT on inflammation and angiogenesis markers

**Figure 5:**
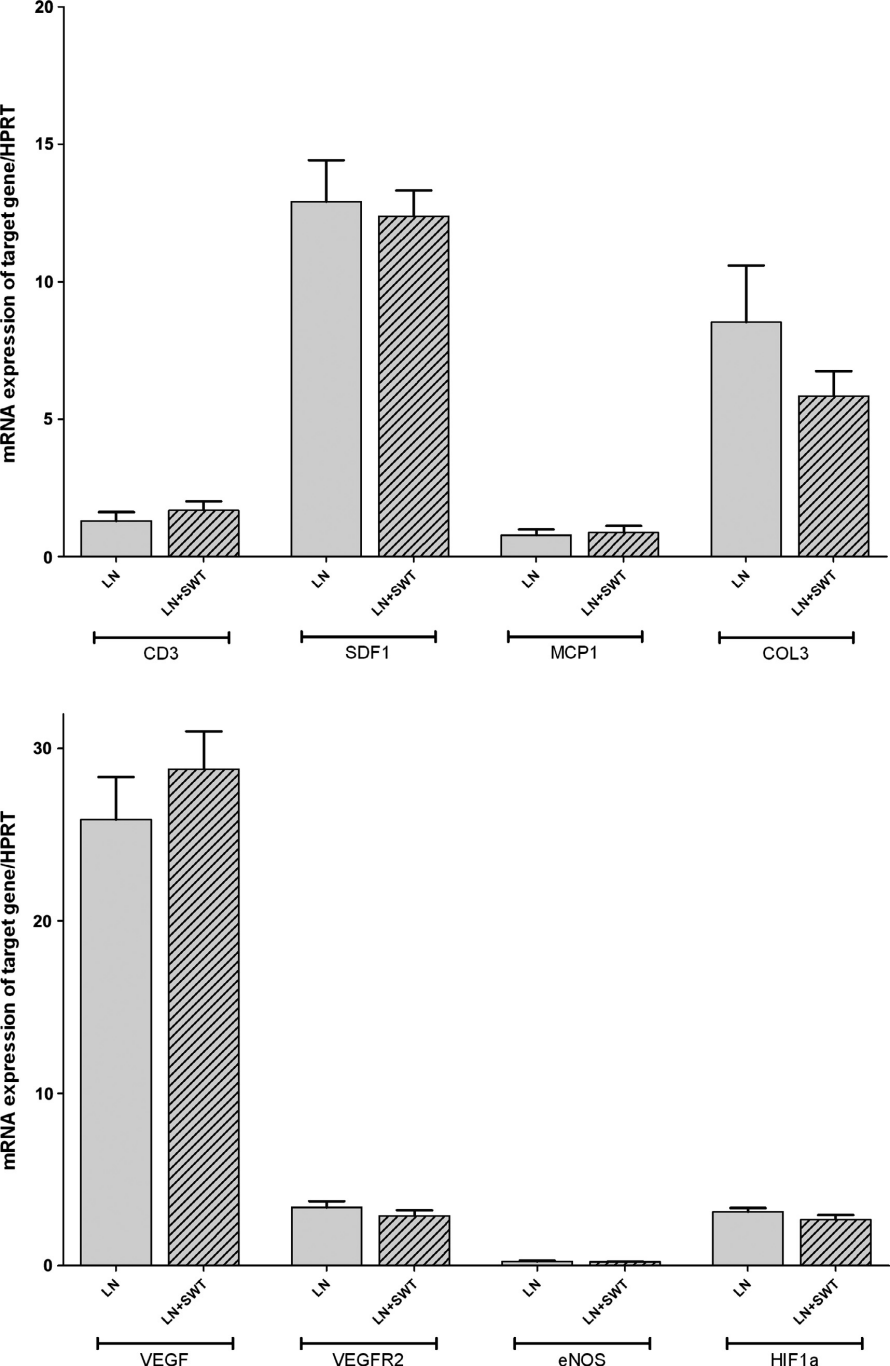
mRNA expression of CD3, SDF‐1, MCP‐1, COL3A1, VEGF, VEGF‐R2, eNOS, and HIF‐1*α* in L‐NAME‐treated + removal rats (LN) and L‐NAME + SWT‐treated rats (LN + SWT) at W5. ^§^No significant differences were observed when rats were treated by SWT.

We quantified mRNA expression of genes involved in nephroangiosclerosis and modified by SWT in previous studies. Expression of CD3, SDF‐1, MCP‐1, markers of inflammation and COL3A1, a marker of fibrosis, was similar between LN and LN + SWT groups (Fig. 5). Moreover, SWT did not increase renal mRNA expression of genes involved in angiogenesis including VEGF, VEGF‐R2, eNOS, and HIF‐1*α* (Fig. 5).

## Discussion

This study investigated for the first time, the impact of SWT on renal repair and angiogenesis in the L‐NAME model of nephropathy. We did not observe any significant improvement of renal repair on top of the beneficial effect of L‐NAME removal. Neither the renal expression of genes involved in angiogenesis (VEGF, VEGF‐R2, eNOS, and HIF‐1*α*), inflammation (CD3, SDF‐1, MCP‐1) and fibrosis (COL3A1), nor the density of peritubular capillaries was increased by the application of SWT.

Chronic treatment with L‐NAME with concomitant administration of NaCl‐induced hypertension and nephroangiosclerosis (Boffa et al. 2003; Ying et al. 2003; Placier et al. 2006). Our results confirmed previous data showing that onset of urinary protein excretion ratio over 1 g/mmol is associated with severe renal lesions (Guerrot et al. 2012). The mean duration of L‐NAME administration was 6 ± 2 weeks. This procedure enabled us to minimize the differences in renal lesions between the animals. After L‐NAME removal, urinary protein excretion decreased progressively despite persistent hypertension. Four weeks after restoration of nitric oxide synthesis, renal lesions improved, but did not normalize, with a decrease in the scores of glomerulosclerosis and arteriosclerosis, and less tubular dilatation and interstitial fibrosis, in keeping with our previous data in mice where we had observed a regression of renal fibrosis 10 weeks after L‐NAME removal (Placier et al. 2006). L‐NAME removal profoundly decreased collagen I gene expression. In contrast to fibrosis scores, peritubular capillaries rarefaction did not recover. Persistent hypertension might prevent vascular healing or the healing process could need a longer time.

In our model, SWT did not ameliorate renal repair and angiogenesis, nor did it change gene expression involved in angiogenesis (VEGF, VEGF‐R2, eNOS, and HIF‐1*α*), inflammation (CD3, SDF‐1, MCP‐1) and fibrosis (COL3A1). Our results contrast to previous experimental and clinical data on myocardial and hind limb ischemia. Indeed, SWT has been shown to improve myocardial dysfunction, hind limb ischemia, and to facilitate cell therapy in patients with chronic heart failure (Assmus et al. 2013; Holfeld et al. 2014). In mini‐pigs submitted to myocardial ischemia and treated by SWT, these beneficial effects were attributed to an increased expression of SDF‐1, CXCR4, eNOS, VEGF genes and vessel density (Fu et al. 2011). SWT effectively reversed ischemia‐induced myocardial dysfunction and remodeling through enhancing angiogenesis (Nishida et al. 2004). During hind limb ischemia in rabbit, SWT was shown to favor the development of collateral arteries and enhance muscle capillary density (Oi et al. 2008). In humans, cardiac shock wave therapy reduced nitro‐glycerine use, improved symptoms of heart failure and myocardial perfusion as assessed by dipyridamole stress thallium scintigraphy (Fukumoto et al. 2006). Moreover, Zeiler A. et al. demonstrated that cardiac shock wave pretreatment with subsequent application of bone marrow‐derived mononuclear cells improved left ventricular ejection fraction in patients with chronic heart failure (Assmus et al. 2013). Preconditioning of the target tissue by shock wave induced upregulation of chemoattractant cytokine SDF‐1 and retention of C‐X‐C chemokine receptor type 4 (CXCR4)‐expressing progenitor cells. However, histological lesions were not assessed except in one study where Masson's trichrome staining was used to analyze fibrosis of ischemic left ventricular myocardium and SWT attenuated myocardial fibrosis (Fu et al. 2011). Studies focused on either cardiac function and/or tissue perfusion by either laser doppler (Assmus et al. 2013), angiography (Nishida et al. 2004; Fu et al. 2011), phletysmograghy (Vardi et al. 2012) or artery blood flow measured by ultrasonic transit‐time flowmeter (Oi et al. 2008).

The lack of therapeutic efficacy of SWT in our case cannot be attributed to the protocol of application of SWT because we scrupulously followed guidelines from Medispec, the provider of the shock wave generator. The targeted kidney was previously localized manually and by ultrasound echography in anesthetized rats in order to focus shock wave on the kidney. Rats were beforehand shaved and a depilatory cream was regularly applied before the implementation of gel to insure an optimal transmission of the shock waves. We could visualize the shock wave across the renal tissue by echography. On the basis of previous studies, we applied a similar protocol with a low energy of shock wave (0.09 mJ/mm^2^, that is about 10% of the energy of lithotripsy treatment), to 2 spots that cover the all kidney with 400 shots/spot. We repeated the SW treatment three times per week for 4 weeks. However, we cannot exclude that a different protocol with a higher number of shoots, longer duration and different energy, might have been more efficient to improve renal repair.

A second hypothesis is a bias related to NO synthases inhibition. The beneficial effects of SW on tissue are probably due to an increased production of nitric oxide by eNOS but also by a nonenzymatic pathway involving the formation of radicals and ions during bubble cavitation (Didenko and Suslick 2002; Gotte et al. 2002; Oi et al. 2008). Based on this physiological effect, Vardi et al. showed in a randomized, double‐blind, sham controlled study that low‐intensity SWT improved clinical erectile dysfunction and penile hemodynamics (Vardi et al. 2012). In our protocol, administration of L‐NAME, a NOS inhibitor, followed by persistent hypertension might have prevented the rise of nitric oxide production induced by shock wave. Similar observations have been made in other studies. In L‐NAME treated and hypertensive rats with left ventricular hypertrophy, cardiac and aorta NOS activity was not restored after 3 weeks L‐NAME cessation (Paulis et al. 2008). Acetylcholine‐induced nitric oxide release in rat renal arterioles was not restored to basal value 2 weeks after L‐NAME removal (Helle et al. 2010). These results suggest that endothelial dysfunction remains after L‐NAME removal and could prevent the angiogenic effect of SWT. It is noteworthy that hypertension was not present in any other previous studies using SWT. A third hypothesis could be the inefficiency of SWT to improve hypertensive nephropathy. Although the kidney as the heart and the muscle can all be ischemic, their respective vasculature, perfusion and global architecture are different, with a more heterogeneous structure of the kidney.

Similar to previous studies, the low‐intensity shock wave energy (0.09 mJ/mm2) was not associated with any side effects such local ecchymosis, macroscopic hematuria. The treatment was well tolerated and all treated animals had similar growth slopes, weight and general looking than untreated rats.

## Conclusion

We were unable to demonstrate that low‐energy shock wave therapy improves renal repair and angiogenesis in a hypertensive nephropathy model. This treatment was safe and well tolerated. The absence of beneficial effect of SWT in L‐NAME‐induced hypertensive nephropathy does not mean that SWT is not efficient in other chronic kidney diseases. Future studies are needed to assess SWT effects in other experimental models as ischemic and diabetic nephropathies.

## Conflict of Interest

None declared.
